# Gallbladder Agenesis with Refractory Choledocholithiasis

**DOI:** 10.1155/2015/747931

**Published:** 2015-06-22

**Authors:** Jamie Tjaden, Kevin Patel, Aziz Aadam

**Affiliations:** Rush University Medical Center, Chicago, IL 60612, USA

## Abstract

Congenital agenesis of the gallbladder is a rare anomaly which is usually asymptomatic and found incidentally. In some cases, however, patients are symptomatic. Common symptoms include right upper quadrant abdominal pain, nausea, and vomiting. Jaundice is present in some symptomatic cases and is due to associated choledocholithiasis (Fiaschetti et al. 2009). In this case, a 63-year-old female presents with jaundice and episodic right upper quadrant abdominal pain with nausea and vomiting. Bilirubin and alkaline phosphatase were found to be markedly elevated. Upper endoscopic ultrasound (EUS) revealed choledocholithiasis, and the patient required multiple endoscopic retrograde cholangiopancreatography (ERCP) sessions before successful extraction of all stones. Subsequent surgical exploration revealed congenital agenesis of the gallbladder. Although this is a rare finding, patients with agenesis of the gallbladder are at increased risk of developing de novo choledocholithiasis which may be challenging to extract.

## 1. Introduction

Congenital agenesis of the gallbladder is a rare anomaly with an average incidence of 0.02% at birth [1]. In 40–70% of cases it is associated with other malformations such as imperforate anus, tracheoesophageal fistula, ventricular septal defect, tetralogy of Fallot, gonadal agenesis, renal dysgenesis, renal agenesis, cleft palate, and cleft lip [2, 3]. The mean age of diagnosis is 46 years, and cases are usually asymptomatic and found incidentally. However, in about 23% of cases, patients become symptomatic. In such patients, right upper quadrant abdominal pain is present in 90% of cases, nausea and vomiting are present in 66%, and jaundice is present in 35% [1]. Choledocholithiasis can be present in 25–50% of cases and may be technically challenging to manage [2].

## 2. Case Description

A 63-year-old female with no significant past medical history presented with a two-month history of jaundice. Over the past year, she experienced four episodes of right upper quadrant abdominal tenderness, pale stools, chills, and vomiting which resolved spontaneously. Her family history is pertinent for two brothers with gallstones. Her physical exam was significant for jaundice and scleral icterus.

The patient's laboratory values were significant for a total bilirubin 7.7 mg/dL, alkaline phosphatase 731 IU/L, AST 151 IU/L, and ALT 68 IU/L. Hepatitis B and hepatitis C, ANA, ASMA, and AMA were negative. Iron studies were normal. Serum ceruloplasmin was slightly elevated.

**Figure 1:**
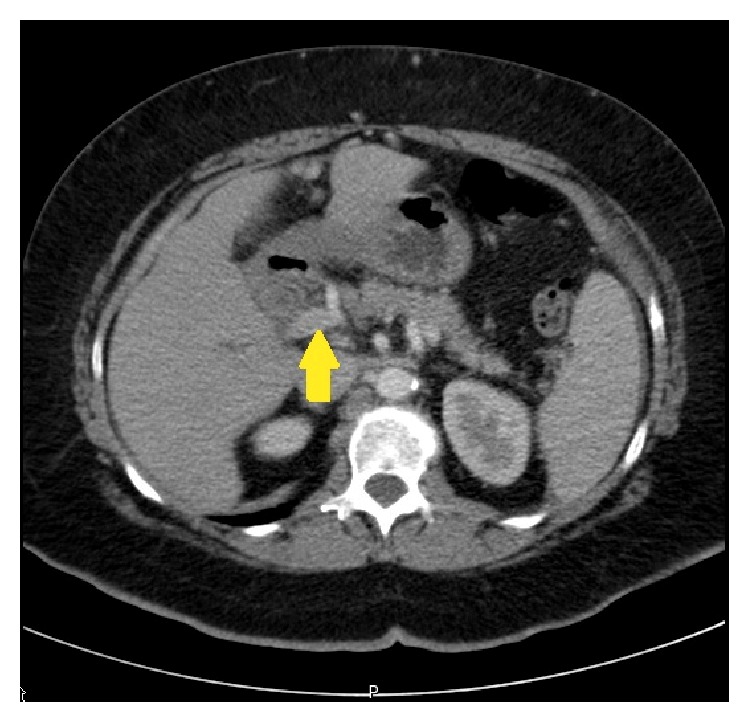
CT abdomen with and without contrast.

**Figure 2:**
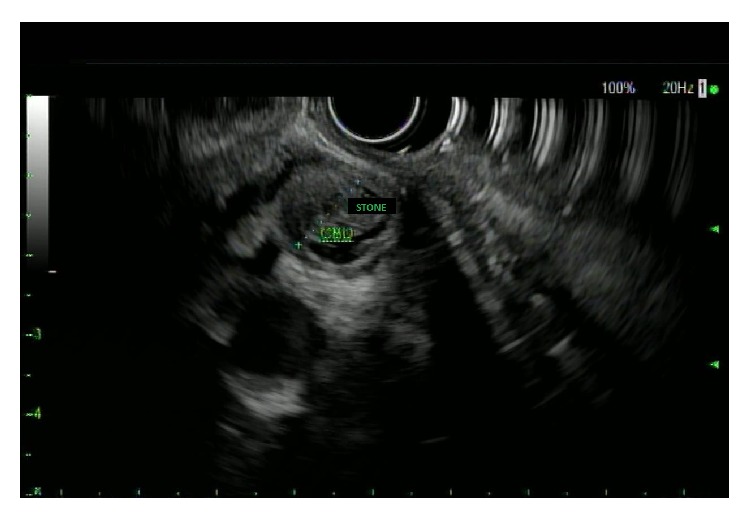
Upper endoscopic ultrasound.

The transabdominal ultrasound revealed a hypoechoic liver, splenomegaly, and what appeared to be a contracted gallbladder with small stones in the absence of common bile duct dilatation. A CT of the abdomen with and without contrast revealed intrahepatic bile duct dilatation, mild distention of the common bile duct, and absence of the gallbladder (Figure 1). EUS revealed hypoechoic material within the common bile duct which was dilated to 11 mm. Posterior acoustic shadowing was seen, suggesting choledocholithiasis (Figure 2).

**Figure 3:**
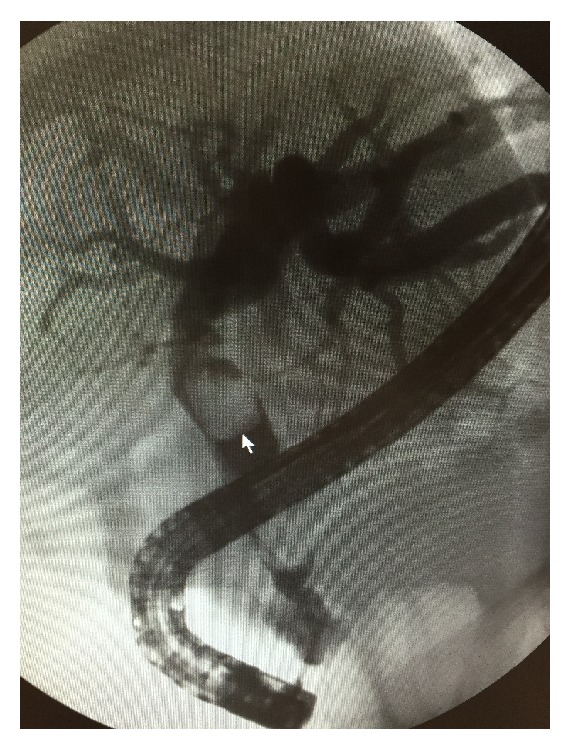
Endoscopic retrograde cholangiopancreatography.

An ERCP was performed and extensive choledocholithiasis was visualized (Figure 3). A sphincterotomy with balloon sweep was performed. Two large 13 mm × 15 mm gallstones could not be cleared from the mid and distal bile duct. After the procedure, LFTs and bilirubin decreased moderately.

One week later, ERCP utilizing mechanical lithotripsy was successful in partial stone extraction. One large stone remained in the mid common bile duct. Liver function tests continued to improve following the procedure. The option of surgical intervention was discussed with the patient, but she preferred the less invasive option of further ERCP treatments as there was progressive improvement in alleviating her stone burden.

Subsequently, ERCP was performed utilizing electrohydraulic lithotripsy (EHL) resulting in minimal clearance of the common bile duct stones. The size of the large stones at the hilum and mid-common bile duct was reduced by 30–50%.

Another ERCP was performed six weeks later utilizing mechanical lithotripsy, but the stones remained impacted in the common bile duct.

After the numerous aforementioned attempts to clear the patient's common bile duct, the decision was made to utilize a more unconventional method. External shock wave lithotripsy (ESWL) was performed with the assistance from a colleague in urology and was successful in complete fragmentation of the remaining stones. This was followed by ERCP with complete removal of the stone fragments.

A few weeks later, a laparoscopic surgery was converted to an open surgery, and the patient was found to have congenital absence of the gallbladder. Intraoperative cholangiogram showed no remaining large stones in the common bile duct. A liver biopsy was taken and revealed mild nonspecific hepatitis and stage 3 bridging fibrosis without cirrhotic nodularity. No stainable iron, copper, or A1AT globules were visualized.

## 3. Discussion

Agenesis of the gallbladder will almost always be misinterpreted by transabdominal ultrasonography as cholecystitis with cystic duct obstruction or as a scleroatrophic gallbladder, therefore leading to unnecessary surgery [1]. This was evident in our case as the patient's choledocholithiasis was misinterpreted as cholelithiasis in a contracted gallbladder. In patients with biliary-type pain and improperly visualized gallbladder on imaging, gallbladder agenesis should be considered.

In agenesis of the gallbladder, 25–50% of patients will develop stones in the common bile duct [2]. The incidence of choledocholithiasis in the general population is unknown but there are over 20 million Americans with gallbladder disease [4] and 5–20% are found to have choledocholithiasis at time of cholecystectomy [5]. The increased frequency of gallstones in patients with gallbladder agenesis has been hypothesized to be due to biliary dyskinesia similar to that seen in postcholecystectomy syndrome. Hypertonic muscular retrograde contraction of the sphincter of Oddi causes biliary dyskinesia leading to common duct dilatation, biliary stasis, and gallstone formation [2].

Conventional therapy for common bile duct stone extraction includes endoscopic sphincterotomy, papillary balloon dilation, and basket and balloon extraction. Conventional therapy is unsuccessful in 10%–15% of cases, usually due to altered anatomy or stones being impacted, large, numerous, barrel or piston-shaped, or in an intrahepatic location.

Advanced therapies include mechanical lithotripsy, electrohydraulic lithotripsy (EHL), laser lithotripsy, and even extracorporeal shock wave lithotripsy (ESWL) in refractory cases. In mechanical lithotripsy, a wire basket is used to capture the stone and then is retracted into a metal sheath, directing a crushing force to the stone. Mechanical lithotripsy has been found to have an overall success rate of 80–90% with 20–30% of cases requiring more than 1 session [6].

In electrohydraulic lithotripsy, there is creation of an electric high-voltage spark between two isolated electrodes located at the tip of a fiber. Sparks are delivered in short pulsations which create immediate expansion of surrounding liquid, inducing an oscillating spherical shock wave which generates pressure to fragment the stone. In one study, 125 patients who failed conventional treatment had a 77% success rate with EHL [7]. In a separate study, 64 of 65 patients were successfully treated with EHL [8].

In laser lithotripsy, a high energy shock wave fragments gallstones as electrons are torn from atomic nuclei. Matter is transformed into a plasma state at the surface of the stone and within adjacent fluid, inducing a spherical shock wave. In one European study with 60 patients refractory to mechanical lithotripsy, laser lithotripsy had an 87% success rate [9]. In one US study with 69 patients, there was a 74% success rate within 1–3 sessions and complete stone clearance in 97% of patients overall [10].

In ESWL, high pressure shock wave energy is focused at a targeted point that traverses the gallstone. Cavitation occurs at the stone surface and changes acoustic impedance, releasing compressive and tensile forces resulting in fragmentation. An ERCP is usually required in order to remove stone fragments. In one study of 60 patients with bile duct stones in whom standard extraction failed, bile duct clearance was achieved in 22 of 30 patients in the ESWL group and in 29 of 30 patients in the laser lithotripsy group (*p* < 0.05). The number of treatment sessions necessary for ESWL was 3.0 ± 1.3 compared to 1.2 ± 0.4 for laser lithotripsy (*p* < 0.001) [11].

Our patient's liver biopsy is also an interesting aspect of this case. The biopsy revealed mild nonspecific hepatitis and stage 3 bridging fibrosis without cirrhotic nodularity. In one study evaluating liver pathology associated with choledocholithiasis, extensive fibrosis with portal-portal linking was present in patients with large duct obstruction and chronic cholestasis. Considerable resolution of fibrosis may still be possible at the stage of portal-portal linking if the obstruction is relieved, which emphasizes the importance of early intervention [12]. It is unknown whether agenesis of gallbladder without a large bile duct obstruction and chronic cholestasis is associated with liver fibrosis.
